# Comparison between high and low potency statins in the incidence of open-angle glaucoma: A retrospective cohort study in Japanese working-age population

**DOI:** 10.1371/journal.pone.0237617

**Published:** 2020-08-17

**Authors:** Nobuhiro Ooba, Rira Iwahashi, Akiko Nogami, Toshimitsu Nakayama, Atsushi Kanno, Naohiro Tochikura, Susumu Ootsuka, Noriyasu Fukuoka

**Affiliations:** 1 Department of Clinical Pharmacy, Nihon University School of Pharmacy, Chiba, Japan; 2 Nihon University Itabashi Hospital Clinical Research Center, Tokyo, Japan; 3 Department of Hospital Pharmacy, Nihon University Itabashi Hospital, Tokyo, Japan; National Eye Institute, UNITED STATES

## Abstract

Some findings on the association between glaucoma and statins in the Asian population have been reported. We conducted a retrospective cohort study using health insurance claims data maintained by the JMDC Inc., which comprises data on about three million individuals representing 2.4% of the Japanese population. The association between the potency of statins and open-angle glaucoma in Japanese working-age population was examined using a commercially available health insurance claims and enrollment database. We identified 117,036 patients with a prescription of statins between January 1, 2005 and March 31, 2014; 59,535 patients were selected as new statin users. Of these, 49,671 (83%) patients without glaucoma who were prescribed statins for the first time were part of the primary analysis. New users of statin were defined as those with a prescription of statin at the beginning of the study, but without a prescription six months earlier. The cohort comprised 29,435 (59%) and 20,236 (41%) patients with a prescription of high-potency statin (atorvastatin and rosuvastatin) and low-potency statin (pravastatin, fluvastatin, pitavastatin, and simvastatin), respectively. Using Cox proportional hazards regression analysis, hazard ratios (HRs) were estimated for glaucoma adjusted for baseline characteristics. Although some baseline characteristics were not similar between the high-potency and low-potency statin groups, the standardized difference for all covariates was less than 0.1. No associations were found between high-potency statin use and glaucoma (adjusted HR = 1.08; 95% confidence interval, 0.93–1.24) in the primary analyses, using the risk for glaucoma in the low-potency statin group as reference. The risk of glaucoma with individual statin use was not significantly different from that with pravastatin. No significant association was found between high-potency statins and the increased risk of glaucoma in Japanese working-age population. Further studies are needed to examine the association between statins and glaucoma in the elderly population.

## Introduction

Statins are widely used as cornerstone cholesterol-lowering drugs to decrease the risk of cardiovascular disease [1]. Regardless of the low-density lipoprotein cholesterol (LDL-C) lowering effect, the cardiovascular protective effect known as the pleiotropic effects is well known for statins [2]. Despite the benefits of statin therapy, intolerance and adverse drug reactions such as myopathy, rhabdomyolysis, elevated hepatic transaminases, and diabetes mellitus have been widely reported [3–4]. Recent studies have shown the association of statin use with a decreased risk of open-angle glaucoma [5–7], while other studies reported differing results regarding the relation of statins to glaucoma incidence [8–10]. The global prevalence of glaucoma is estimated at 3.5% in the 40- to the 80-year old population [11], and it is one of the risk factors for loss of visual function [12]. The five main causes of visual impairment in Japan are glaucoma (24.3%), diabetic retinopathy (20.6%), degenerative myopia (12.2%), age-related macular degeneration (10.9%), and cataract (7.2%) [13]. Therefore, it is essential to assess the possible risk of glaucoma in hyperlipidemia patients undergoing treatment with statins.

The findings on the association between statins and glaucoma are inconsistent. Continued use of statin for 2 years decreased the risk of developing open-angle glaucoma [adjusted hazard ratio (HR), 0.92; 95% confidence interval (CI), 0.87 to 0.98] [5]. A population-based case-control study in Taiwan reported that while high-dose statin use elevated the open-angle glaucoma incidence [adjusted odds ratio (OR), 1.24; 95% CI, 1.03 to 1.49], statin use showed no association (adjusted OR, 1.02; 95% CI, 0.90 to 1.15) [8]. Additionally, Talwar et al. reported that continuous use of statins for 2 years had a 21% reduced risk of glaucoma compared with no use (adjusted HR, 0.79; 95% CI, 0.66–0.96) [7]. The protective effect of different statins on open-angle glaucoma was not significantly different; the HR ranged from 0.61 to 1.29 when compared to atorvastatin as a reference [7]. Further, statin use was associated with higher intraocular pressure (0.21 mmHg higher; 95% CI, 0.02 to 0.04 mmHg), which is the only modifiable risk factor for glaucoma [14]. Though the lipid-lowering effect on low-density lipoprotein cholesterol (LDL-C) is different for different statins, it is unclear whether the risk of glaucoma is similar between them. Further, the study size of previous reports [8,14] on the association between statins and the incidence of glaucoma in Asian populations was not remarkably large. Therefore, we conducted a retrospective cohort study using health insurance claims data to assess the association between statins and glaucoma in Japanese working-age population.

## Materials and methods

### Data sources

We used commercially available health insurance claims and enrollment data of approximately three million individuals, maintained by the JMDC Inc. (Tokyo, Japan) [15], between January 1, 2005 and March 31, 2014 [16]. The JMDC database represents about 2.4% of the Japanese population as of 2014 and the data is anonymized. Data on beneficiaries in the 20- to 74-year age group who were workers in private firms along with their dependents, including the inpatient and outpatient claims in hospitals, and the dispensing claims of pharmacies were included. The insurance claims data contained information regarding the use of health care services, diagnoses, medical procedures, and the use of drugs. As the inpatient claims have the same format as the outpatient claims in Japan, the information concerning drug use during hospitalization was obtained from the inpatient claims. The claims database did not contain data of those aged 75 years and older as they were not covered by corporate health insurance but by the public health insurance (late-stage medical care system for the elderly) for all Japanese citizens aged 75 or older [17]. The enrollment data contained the year of birth, sex, and the dates of enrollment and disenrollment of the insured. In the claims data, more than 20,000 local drug codes were used to specify the trade name and generic name for all the approved drugs [18]. The generic name for drugs was coded by the Anatomical Therapeutic Chemical (ATC) code [19] and the diagnoses were coded using the International Classification of Diseases, 10^th^ revision (ICD-10) [20].

### Study cohort and inclusion criteria

**Fig 1:**
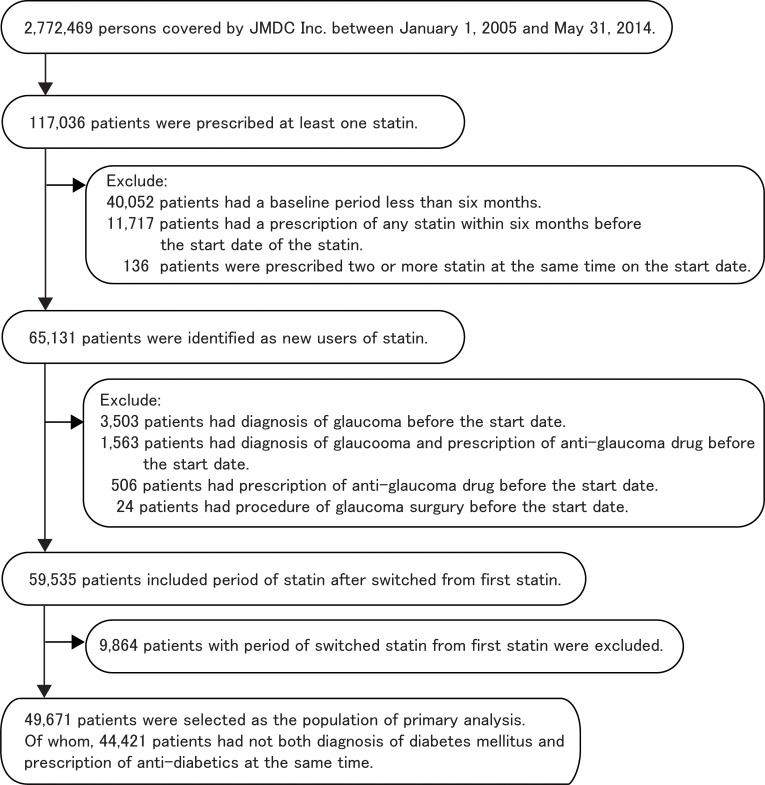
Flow chart of the study population.

We identified 117,036 patients with at least one claim of any statin use during the study period (Fig 1). The index date of the study period was defined as the date of starting statin use. The baseline period was defined as the period when the data on claims was available, after the date of enrollment but before the index date. To select new statin users, we excluded patients with a baseline period less than six months, a diagnosis of glaucoma (H40 in ICD-10 code), use of any drug for glaucoma, or if there was a record of any procedure for glaucoma before the index date. Patients with a prescription of statin six-months before the index date were also excluded. Patients were also excluded if two or more statins were prescribed at the same time on the index date. In the sensitivity analysis, if a patient was prescribed a different statin after six months of stopping the first statin, the patient was included as a new user of statin. In addition, if a patient with new statin use switched to another statin, the patient was included.

### Exposures, outcomes, and covariates

Since the LDL-C lowering effect of different statins is different, more than 50% with atorvastatin and rosuvastatin and 30% to 50% reduction with pravastatin, fluvastatin, pitavastatin, and simvastatin) [2], the potency of statins may affect the incidence of glaucoma. According to a previous study [21], the statins were classified as ‘low potency statin’ (pravastatin, fluvastatin, pitavastatin, and simvastatin), and ‘high potency statin’ (atorvastatin and rosuvastatin); however, data on the daily dose of statins was not available.

Glaucoma in a patient was identified based on the diagnosis code of glaucoma (ICD-10 codes for H401, H406, and H409), a domestic procedure code of glaucoma surgery, or if a drug for glaucoma (S01E2 coded by ATC code) was used after the index date.

The following covariates obtained from the claims data were considered as confounders and were adjusted for in the analysis: age, sex, use of other prescribed medications [antidiabetic drugs, antihypertensives, antidepressants, benzodiazepines, anticoagulants, antiplatelet drugs, aspirin, antigout drugs, and non-statins (including fibrates, ezetimibe, and nicotinic acid)], and diagnosis of certain diseases (diabetes mellitus, hypertension, myocardial infarction, chronic heart failure, cerebrovascular disease, renal disorders, liver disorders, pulmonary disease, and cancer). The covariates benzodiazepines, antigout drugs, renal disorders, and liver disorders were included because previous studies have reported that benzodiazepines are associated with acute angle-closure glaucoma [22], serum uric acid levels are associated with primary open-angle glaucoma [23], and nonalcoholic fatty liver disease [24] and hemodialysis [25] are associated with elevated intraocular pressure. The Charlson comorbidity index [26] for prediction of mortality was also calculated using ICD-10 code and included as a covariate. These covariates were measured during the baseline period of 6 months before the first prescription of a statin.

### Statistical analysis

To describe the baseline characteristics, we assessed the demographics, co-medications, and comorbidity in the new users of statin during the six months before the index date. To assess the distribution of potential confounders at baseline in the high-potency and low-potency groups, we estimated P-values (t-test) and the standardized difference; a value greater than 0.1 was considered as a meaningful difference [27]. In the primary analysis, we defined the observation period for assessment of the outcome as follows: the period from the index date to the incidence date of glaucoma, date of switching or stopping the statin, or disenrollment date or March 31, 2014, whichever was earlier. We censored the observation period when the period between the prescription dates of statins was greater than 30 days. Cox proportional hazards regression model was used to estimate the effect of unadjusted and adjusted models of low-potency statins and high-potency statins on glaucoma. The model incorporated into listed covariates is shown in Table 1. In the analyses, we used the incidence rate with the low-potency statin as the reference. Furthermore, we estimated the effect of the individual statins on the incidence of glaucoma using the primary dataset to compare the effect of pravastatin on the incidence of glaucoma.

**Table 1:** Baseline characteristics of the study cohort categorized by potency of statins.

	High-potency statins (atorvastatin, rosuvastatin), N = 29,435	Low-potency statins (pravastatin, fluvastatin, pitavastatin, simvastatin) N = 20,236	Standardized difference^b^	P-value
Age (years), Mean ± SD	51.2 ± 9.7	51.6 ± 9.9	-0.036	<0.001
Age groups, N (%)				
<40 years	3,899 (13.2)	2,692 (13.3)		-
40 to 49 years	8,753 (29.7)	5,569 (27.5)		-
50 to 59 years	11,218 (38.1)	7,905 (39.1)		-
≥60 years	5,565 (18.9)	4,070 (20.1)		-
Males, N (%)	18,364 (62.4)	11,715 (57.9)	0.092	<0.001
Mean follow-up days	282.2	272.0	-	
Co-medications, N (%)				
Antidiabetics	3,233 (11.0)	2,146 (10.6)	0.012	0.181
Antihypertensives	9,532 (32.4)	6,459 (31.9)	0.010	0.276
Antidepressants	1,174 (4.0)	808 (4.0)	-0.0002	0.980
Benzodiazepines	3,826 (13.0)	2,778 (13.7)	-0.022	0.019
Anticoagulants	375 (1.3)	241 (1.2)	0.008	0.408
Antiplatelet drugs	1,376 (4.7)	694 (3.4)	0.063	<0.001
Aspirin	1,907 (6.5)	1,158 (5.7)	0.032	0.001
Anti-gout drugs	2,415 (8.2)	1,650 (8.2)	0.002	0.839
Non-statins^a^	1,620 (5.5)	1,042 (5.1)	0.016	0.083
Comorbidities, N (%)				
Diabetes mellitus	10,859 (36.9)	7,349 (36.3)	0.012	0.191
Hypertension	11,127 (37.8)	7,680 (38.0)	-0.003	0.735
Chronic heart failure	2,131 (7.2)	1,317 (6.5)	0.029	0.002
Cerebrovascular disease	3,230 (11.0)	2,108 (10.4)	0.018	0.048
Myocardial infarction	1,057 (3.6)	638 (3.2)	0.024	0.008
Liver disease	3,981 (13.5)	2,825 (14.0)	-0.013	0.167
Renal disease	1,346 (4.6)	944 (4.7)	-0.004	0.630
Pulmonary disease	3,448 (11.7)	2,508 (12.4)	-0.021	0.023
Cancer	4,173 (14.2)	2,945 (14.6)	-0.011	0.240
Charlson Comorbidity Score (Mean ± SD)	1.6 ± 2.1	1.6 ± 2.1	0.027	0.003

SD, standard deviation

^a^ Non-statins contained fibrates, ezetimibe, and nicotinic acid

^b^ Standardized difference value greater than 0.1 was considered meaningful [27]

We conducted several sensitivity analyses. Firstly, patients with diabetes mellitus as a risk factor for glaucoma [28] were excluded from the study considering the effect of residual confounding as well as the results of a study by Kreft et al. [29]. Secondly, our analysis included the period after switching the statins, although the observation period was censored when a statin was discontinued or switched in the primary analysis. In this sensitivity analysis, we used the latest value of the covariates assuming that they were not affected by prior statin use when a different statin was prescribed. In this case, we calculated the robust variance to estimate the effect. Thirdly, we limited the study population to those over the age of 40 years considering that those aged less than 40 years may not be at risk for glaucoma [30]. Lastly, considering the hydrophilic (pravastatin and rosuvastatin) or lipophilic (atorvastatin, fluvastatin, pitavastatin, and simvastatin) property of statins [2], we estimated the HR and its 95% CI for glaucoma. All analyses were performed with SAS 9.4 (SAS Institute Inc., Cary NC, USA). A P-value of <0.05 was considered statistically significant. This study was approved by the Ethics Committee of the Nihon University School of Pharmacy, which waived the requirement for obtaining informed consent.

## Results

We identified 117,036 patients with a prescription of statin during the study period, and 59,535 patients were enrolled as new statin users in the study. Of these, 49,671 (83%) patients without glaucoma were users with a first-time prescription for statin; they were included in the primary analysis (Fig 1). The study cohort comprised 29,435 (59%) patients with a prescription for high-potency statin [atorvastatin (n = 13,305) and rosuvastatin (n = 16,130)] and 12,064 (24%) patients with a prescription for low-potency statin [pravastatin (n = 8,718), fluvastatin (n = 1,476), pitavastatin (n = 8,172), and simvastatin (n = 1,870)]. The baseline characteristics of the study cohort are shown in Table 1. Though the baseline characteristics were not similar between the high-potency and low-potency statin group regarding some co-medications and comorbidities based on the P-value (<0.05), none of the standardized difference values exceeded 0.1.

Compared to the risk of glaucoma in the low-potency statin group, the unadjusted, age-sex adjusted, and multivariable-adjusted HRs for high-potency statin group are shown in Table 2. The risk of glaucoma in the high-potency statin group was similar to that of the low-potency statin group (HR = 1.08; 95% CI, 0.93–1.24) in the primary analysis. In the sensitivity analysis with different outcomes, the effect of high-potency statins on glaucoma was non-significant compared to low-potency statins.

**Table 2:** Cox proportional hazards regression model to estimate the effect of unadjusted and multivariate-adjusted models of low-potency statins and high-potency statins on glaucoma incidence.

	Low-potency statins (pravastatin, fluvastatin, pitavastatin, simvastatin)	High-potency statins (atorvastatin, rosuvastatin)
Number of patients	20,236	29,435
Outcome		
Number of patients with diagnosis of glaucoma or used anti-glaucoma drugs	308	493
Hazard ratio (95% confidence interval) and P = value		
Unadjusted	1.0	1.06 (0.92–1.22), P = 0.45
Age and sex adjusted	1.0	1.08 (0.94–1.25), P = 0.28
Multivariate^a^ adjusted	1.0	1.08 (0.93–1.24), P = 0.31
Outcome		
Number of patients with diagnosis of glaucoma	260	422
Hazard ratio (95% confidence interval) and P-value		
Unadjusted	1.0	1.07 (0.92–1.25), P = 0.37
Age and sex adjusted	1.0	1.10 (0.94–1.29), P = 0.23
Multivariate^a^ adjusted	1.0	1.09 (0.94–1.28), P = 0.26
Outcome		
Number of patients using anti-glaucoma drugs	108	172
Hazard ratio (95% confidence interval) and P-value		
Unadjusted	1.0	1.05 (0.83–1.34), P = 0.67
Age and sex adjusted	1.0	1.09 (0.86–1.39), P = 0.48
Multivariate^a^ adjusted	1.0	1.09 (0.85–1.39), P = 0.50

^a^ Adjusted for age, sex, co-medications (antidiabetic drugs, antihypertensive drugs, antidepressants, benzodiazepines, anticoagulants, antiplatelet drugs, aspirin, anti-gout agents, and non-statins), comorbidities (diabetes mellitus, hypertension, chronic heart failure, cerebrovascular disease, myocardial infarction, liver disease, renal disease, pulmonary disease, and cancer), and Charlson comorbidity score

We could not estimate the HR for glaucoma surgery in the high-potency statin group as only two patients in the low-potency statin group had the procedure code and the use of anti-glaucoma drugs simultaneously.

Table 3 shows the comparison of HR of individual statins with the glaucoma incidence in pravastatin users. The risk for glaucoma between the statins was similar.

**Table 3:** Association between individual statins and glaucoma incidence.

Statin	Number of patients	Number of patients with diagnosis of glaucoma or used anti-glaucoma drugs	Hazard ratio (95% confidence interval) and P-value		
			Unadjusted	Age and sex adjusted	Multivariate^a^ adjusted
Pravastatin	8,718	138	1.00	1.00	1.00
Fluvastatin	1,476	17	0.71 (0.43–1.17), P = 0.18	0.71 (0.43–1.17), P = 0.18	0.70 (0.42–1.16), P = 0.17
Pitavastatin	8,172	133	1.03 (0.81–1.30), P = 0.83	1.06 (0.84–1.35), P = 0.63	1.01 (0.80–1.29), P = 0.91
Simvastatin	1,870	20	0.80 (0.50–1.28), P = 0.34	0.80 (0.50–1.28), P = 0.34	0.81 (0.50–1.29), P = 0.37
Atorvastatin	13,305	237	1.09 (0.88–1.34), P = 0.45	1.12 (0.91–1.39), P = 0.28	1.10 (0.89–1.36), P = 0.40
Rosuvastatin	16,130	256	0.98 (0.80–1.21), P = 0.85	1.02 (0.83–1.26), P = 0.84	1.00 (0.81–1.23), P = 0.99

^a^ Adjusted for age, sex, co-medications (antidiabetic drugs, antihypertensive drugs, antidepressants, benzodiazepines, anticoagulants, antiplatelet drugs, aspirin, anti-gout agents, and non-statins), comorbidities (diabetes mellitus, hypertension, chronic heart failure, cerebrovascular disease, myocardial infarction, liver disease, renal disease, pulmonary disease, and cancer), and Charlson comorbidity score

The sensitivity analyses were conducted in patients without diabetes mellitus, in the patients who had switched to another statin, or in those aged 40 years or older (Table 4). No significant difference was observed when the diagnosis of glaucoma or the use of anti-glaucoma drugs were defined as the outcomes compared to the risk of glaucoma with low-potency statins. Further, the risk for glaucoma between hydrophilic statins and lipophilic statins was similar (HR, 0.97; 95% CI, 0.85–1.12).

**Table 4:** Adjusted risk for glaucoma between statin groups in sensitivity analyses.

	Adjusted hazard ratio (95% confidence interval) and P-value for Outcomes		
	Diagnosis of glaucoma	Use of anti-glaucoma drugs	Diagnosis of glaucoma or use of anti-glaucoma drugs
Without diabetes mellitus^a^			
Low-potency statins (pravastatin, fluvastatin, pitavastatin, simvastatin)	1.0	1.0	1.0
High-potency statins (atorvastatin, rosuvastatin)	0.99 (0.83–1.17), P = 0.86	1.08 (0.82–1.42), P = 0.60	0.99 (0.85–1.16), P = 0.93
In users after switching to another statin^b^			
Low-potency statins (pravastatin, fluvastatin, pitavastatin, simvastatin)	1.0	1.0	1.0
High-potency statins (atorvastatin, rosuvastatin)	1.07 (0.92–1.23), P = 0.77	1.07 (0.85–1.34), P = 0.58	1.06 (0.93–1.21), P = 0.40
40 years or older^b^			
Low-potency statins (pravastatin, fluvastatin, pitavastatin, simvastatin)	1.0	1.0	1.0
High-potency statins (atorvastatin, rosuvastatin)	1.10 (0.93–1.29), P = 0.26	1.08 (0.84–1.39), P = 0.53	1.08 (0.93–1.25), P = 0.34
Based on statin property^b^			
Lipophilic statins (atorvastatin, fluvastatin, pitavastatin, simvastatin)	1.0	1.0	1.0
Hydrophilic statins (pravastatin, rosuvastatin)	0.95 (0.81–1.10), P = 0.48	1.10 (0.87–1.39), P = 0.45	0.97 (0.85–1.12), P = 0.72

^a^ Adjusted for age, sex, co-medications (antihypertensive drugs, antidepressants, benzodiazepines, anticoagulants, antiplatelet drugs, aspirin, anti-gout agents, and non-statins), comorbidities (hypertension, chronic heart failure, cerebrovascular disease, myocardial infarction, liver disease, renal disease, pulmonary disease, and cancer), and Charlson comorbidity score

^b^ Adjusted for age, sex, co-medications (antidiabetic drugs, antihypertensive drugs, antidepressants, benzodiazepines, anticoagulants, antiplatelet drugs, aspirin, anti-gout agents, and non-statins), comorbidities (diabetes mellitus, hypertension, chronic heart failure, cerebrovascular disease, myocardial infarction, liver disease, renal disease, pulmonary disease, and cancer), and Charlson comorbidity score

## Discussion

In this retrospective cohort study, the risk for glaucoma was similar between the high-potency statin group (atorvastatin and rosuvastatin) and the low-potency statin group (pravastatin, fluvastatin, pitavastatin, and simvastatin). Additionally, the adjusted HR of glaucoma between different statins was also similar. Regardless of the potency of statins, the effect of different statins on the risk of glaucoma was not different as the findings of the primary and the sensitivity analyses (considering the hydrophilic or lipophilic properties) remained consistent.

The protective effect of statins on glaucoma has been reported in most of the previous studies [5–7, 31,32]; however, some studies have also found that the use of statins is not associated with glaucoma [8–10]. The control groups in these studies were not active controls like statin users; rather, the controls were non-statin users. As the potency or property of statin differs between different statins [2], it is important to clarify whether the protective effect for glaucoma is a class effect of statin or the effect of a specific statin.

Our findings are consistent with the previous study by Talwar et al. who also used insurance claims data of managed care in the United States [7]. In their study, the protective effect (HR) of individual statins (lovastatin, cerivastatin, rosuvastatin, fluvastatin, pravastatin, and simvastatin) for open-angle glaucoma was not significantly different than that of atorvastatin. A study by Chen et al., also reported no association between any subtype statin and the risk of open-angle glaucoma, compared to that in non-users of statin [8]. Similar to previously stated studies [7–8], our results show that high-potency statins and lipophilic statins are not associated with significantly different incidence of open-angle glaucoma compared to low-potency statins and hydrophilic statins, respectively. Furthermore, the various statins are not associated with significantly different incidence of open-angle glaucoma compared to pravastatin.

Diabetes mellitus is a well-known risk factor of glaucoma [28]. One of our sensitivity analyses was conducted in patients without diabetes mellitus [29] as the proportion of those with a diagnosis of diabetes (p = 0.01) or use of antidiabetics (p = 0.01) at baseline was significantly different between the high-potency and low-potency statin groups; the standardized difference between these was less than 0.1. The results of the sensitivity analysis were similar to that of the primary analysis.

There are several strengths of our study. First, we used a new-user cohort design to prevent prevalent user bias. Second, to mitigate the effect of confounding factors by indication, we conducted a sensitivity analysis in patients without diabetes mellitus. Third, to the best of our knowledge, our study size was larger than those of previous studies [8,14,32] on the association between statin use and glaucoma in the Asian population, even though diabetes, which is a risk factor for glaucoma, is rapidly increasing in some Asian countries [33].

Our study has some limitations. Firstly, the diagnosis code for glaucoma in our claims database was not validated. The outcome of glaucoma is likely of mixed etiology (such as open-angle glaucoma, exfoliative glaucoma, some secondary glaucoma, and some ocular hypertension). This nondifferential misclassification may have biased the associations towards null. If the definition of the outcome included confirmed cases of primary open-angle glaucoma with visual field loss, the possibility of misclassification would have been mitigated, thus lowering the bias. However, our claims database did not contain data on visual field loss. To cover maximum patients suffering from glaucoma, the study outcome in our primary analysis was defined as a diagnosis code of glaucoma or the use of anti-glaucoma drugs. Only those with a diagnosis for glaucoma and the use of a drug to lower intraocular pressure for glaucoma alone were examined separately. Although these findings were consistent, some of the anti-glaucoma drug users may have been patients with ocular hypertension without glaucoma. The users of anti-glaucoma drugs are likely to be patients with glaucoma, including those with normal-tension glaucoma who needed to reduce the intraocular pressure [34]. However, Gordon et al. reported that 22% of the patients using anti-glaucoma drugs for ocular hypertension developed primary open-angle glaucoma [35]. Secondly, although high or low dosages may confound the definition of high- or low-potency statins, we could not examine the dose-response relationship between statins and glaucoma. In a population-based case-control study using a claims database in Taiwan [8], high-dose statin use (≥120 DDD/y) was associated with a 1.26-fold increase in the risk of open-angle glaucoma compared with no statin use. If a high dose of statin affects the incidence of glaucoma, then a higher potency of statin may also have a similar effect. However, neither our study nor the study in Taiwan showed any such effect on the risk of glaucoma. Thirdly, our study population did not include the elderly population (75 years or older), although a statin-continuous user with hyperlipidemia over 60 years contributed to the 8% decreased risk of open-angle glaucoma [5]. Glaucoma frequently presents in the elderly [36]. The reasons are unknown but the effect of statin on glaucoma may differ in the elderly population [5,7]. Further research on this aspect is required. On the other hand, in the sensitivity analysis after excluding those less than 40 years of age, the results were consistent with those of the primary analysis. Fourthly, we could not obtain data on the elevation of intraocular pressure [37], body mass index, LDL-C, blood pressure, fasting blood glucose, and visual field loss. As a result, there could be residual confounding. Fifthly, there might have been some detection bias in that the glaucoma is an insidious disease and it is possible that those who are prescribed statins are more likely to be diagnosed with glaucoma. Therefore, there may be a bias towards the null even if there was a true inverse association with high-potency statin use. Lastly, we could not examine the biological mechanism between statin and glaucoma due to the lack of data on LDL-C, which did not allow us to take the statin associations to the biological context.

## Conclusions

The use of the high-potency statins was not significantly associated with glaucoma incidence in a Japanese working-age population with low-potency statins as the reference. Additionally, the risk of glaucoma between individual statins was similar. Further studies are needed to explore the association between glaucoma and statins in the elderly and to clarify the risk with non-statin use.
